# Description of a new dagger nematode, *Xiphinema barooghii* n. sp. (Nematoda: Longidoridae) and additional data on the three known species of the genus from northwest of Iran

**DOI:** 10.21307/jofnem-2019-007

**Published:** 2019-04-16

**Authors:** Nasir Vazifeh, Gholamreza Niknam, Habibeh Jabbari, Arezoo Naghavi

**Affiliations:** 1 Department of Plant Protection, Faculty of Agriculture, University of Tabriz, Tabriz, Iran; 2 Department of Plant Protection, Faculty of Agriculture, University of Maragheh, Maragheh, Iran

**Keywords:** D2–D3 of 28S rDNA, Longidorids, Molecular analysis, Morphology, Morphometrics, New species, Taxonomy, *Xiphinema index*, *X. pachtaicum* and *X. vuittenezi*

## Abstract

*Xiphinema barooghii* n. sp. collected from the rhizosphere of common wheat (*Triticum aestivum* L.) in Roodghat area, Sufiyan, East-Azarbaijan province, northwest of Iran, is described on the basis of the morphological, morphometric and molecular data. The new species belongs to morphospecies group 6 of the polytomous key prepared by Loof and Luc, 1990. *Xiphinema barooghii* n. sp. is characterized by having two almost equally developed female reproductive branches with spines in the tubular portion of the uterus, a body length of 3.67–4.25 mm, a flat lip region, rounded cephalic region, separated from body contour by a shallow depression, a spear 215–225 μm long, mid-body diameter of 60–79 μm, vulva (46–48%), a short tail (30–38 µm, c = 103–133, c′ = 0.7–0.9), conoid, dorsally convex, ventrally directed with a small terminal peg and a distinct terminal blind canal, the presence of four juvenile stages and the absence of males. The polytomous identification codes of the new species are: A4, B3, C5a, D6, E5, F4, G3, H2, I3, J4, K2, L1. In addition to morphological and morphometric data, molecular analyses of the D2–D3 expansion regions of the 28S rDNA gene placed the new species as a sister species of *X. herakliense* (Group 5) with 65% Bayesian posterior probability and further separated this species from the other members in group 6. In this study, *X. index*, *X. pachtaicum* and *X. vuittenezi* were also collected and additional data for the species were provided.

The genus *Xiphinema*, excluding the *X. americanum* group, comprises a complex of more than 220 species divided into eight morphospecies groups (Loof and Luc, 1990), based on the structural diversity of the female reproductive system, body habitus, lip region shape, total spear, body length and tail shape of female and juveniles using a polytomous key. Within the proposed groups, members of the group 6 (*X. pyrenaicum* species complex) are characterised by having two equally developed female genital branches with the presence of spines in the tubular part of the uterus and a rounded tail with or without a conspicuous projecting bulge.

According to Ghaderi et al. (2018), 26 species of the genus *Xiphinema* have been reported from Iran. Later on, some other species including *X. simile* (Lamberti et al., 1983; Naghavi et al., 2018), *X. macroacanthum* (Lamberti et al., 1989), and *X. utahense* (Lamberti and Bleve-Zacheo, 1979; Jabbari et al., 2018) have also been added to our country’s nematode fauna.

In a recent nematological survey on longidorids in Roodghat area, Sufiyan, East-Azarbaijan province in northwest of Iran, three known and one new species of the genus *Xiphinema* were found in several agroecosystems. The new species belonging to morphospecies group 6 of the genus was recovered from soil samples collected from the rhizosphere of common wheat and is described herein as *X. barooghii* n. sp. This is the fifth species belonging to morphospecies group 6 originally described from Iran, the other species being *X. robbinsi* (Pedram et al., 2008), *X. iranicum* (Pedram et al., 2009), *X. mazandaranense* (Pedram, Pourjam, Robbins, Ye, Atighi, and Decramer, 2012; Pedram, Pourjam, Palomares-Rius, Ghaemi, Cantalapiedra-Navarrete and Castillo, 2012) and *X. zagrosense* (Ghaemi et al., 2012), and except for *X. robbinsi*, the remaining three species have only been reported from Iran.

## Materials and methods

### Sampling, extraction, mounting, and drawing

During a survey on nematode diversity in agroecosystems of Roodghat area, Sufiyan, East- Azarbaijan province in northwest of Iran, several soil samples were collected during 2016 and 2017 and processed at the Nematology Laboratory, University of Tabriz, Tabriz, Iran. The samples were taken from a depth of 5–30 cm. Nematodes were extracted using a tray (Whitehead and Hemming, 1965) and posteriorly killed, fixed and transferred to anhydrous glycerin using the method proposed by De Grisse (1969). Temporary and permanent microscopic slides of the nematodes were prepared to study morphological and morphometric characters. Morphometric data were obtained using a drawing tube attached to an Olympus BX41 light microscope. Photographs were taken by a DP50 digital camera system connected to the microscope. Raw photographs were edited using Adobe® Photoshop® CS. Drawings were made by Corel DRAW®, software version 12.

### DNA extraction, PCR, and sequencing

For DNA extraction from *X. barooghii* n. sp. and *X. index*, a live adult nematode from each was handpicked and separately placed in a small drop of distilled water or worm lysis buffer on a clean slide and crushed by a sterilized scalpel. The suspension was transferred to an eppendorf tube containing 25.65 μl ddH2O, 2.85 μl 10x PCR buffer and 1.5 μl proteinase K (600 μg/ml) (Promega, Benelux, The Netherlands). The tubes were stored at −80°C for 1 hr, incubated at 65°C for 1 hr and heated for 15 min at 95°C to inactivate the proteinase. The DNA sample was stored at −20°C until used as the PCR template. The D2–D3 domain region of LSU was amplified with the forward primer D2A (5′-ACAAGTACCGTGAGGGAAAGTTG-3′) and the reverse primer D3B (5′-TCGGAAGGAACCAGCTACTA-3′) (Nunn, 1992). The 25 μl PCR reaction mixture was composed by 10 μl ddH2O, 12.5 μl PCR master mix (Ampilliqon, Denmark), 0.75 μl of each forward and reverse primers and 1 μl of DNA template. The process was carried out using a Thermocycler Machine in accordance with Archidona-Yuste et al. (2016). PCR cycle conditions were as follows: denaturation at 94°C for 2 min, 35 cycles of denaturation at 94°C for 30 s, annealing of primers at 55°C for 45 s and extension at 72°C for 3 min followed by a final elongation step at 72°C for 10 min. PCR products were purified and sequenced by the Applied Biosystems® 3730/3730xl DNA Analyzer in South Korea. The recently obtained sequences of the new species and *X. index* were deposited in GenBank database under accession numbers MH884067 for *X. barooghii* n. sp. and MH879782 for *X. index* as indicated on the phylogenetic tree (Fig. 5).

### Phylogenetic analyses

The newly obtained sequences were aligned using MEGA6 (Tamura et al., 2013) and compared with other *Xiphinema* D2–D3 expansion segment of 28S rDNA gene sequences available in GenBank using the Nblast homology search program. *Longidorus helveticus* (Lamberti et al., 2001) (AY601566) was chosen as out group. The best-fitted model of DNA evolution was obtained using MrModeltest 2.3 (Nylander, 2004) with the Akaike Information Criterion (AIC). Phylogenetic analysis of the sequence data sets was performed based on Bayesian Inference algorithm implemented in MrBayes 3.1.2 (Ronquist and Huelsenbeck, 2003) under the general time-reversible model with invariable sites and a gamma-shaped distribution (GTR + I + G). After discarding burn-in samples and evaluating convergence, the remaining samples were retained for further analyses. Posterior probabilities (PP) are given on appropriate clades. Tree was visualised using Fig tree 1.4.3 v.

## Results

### Systematics

**Figure 1:**
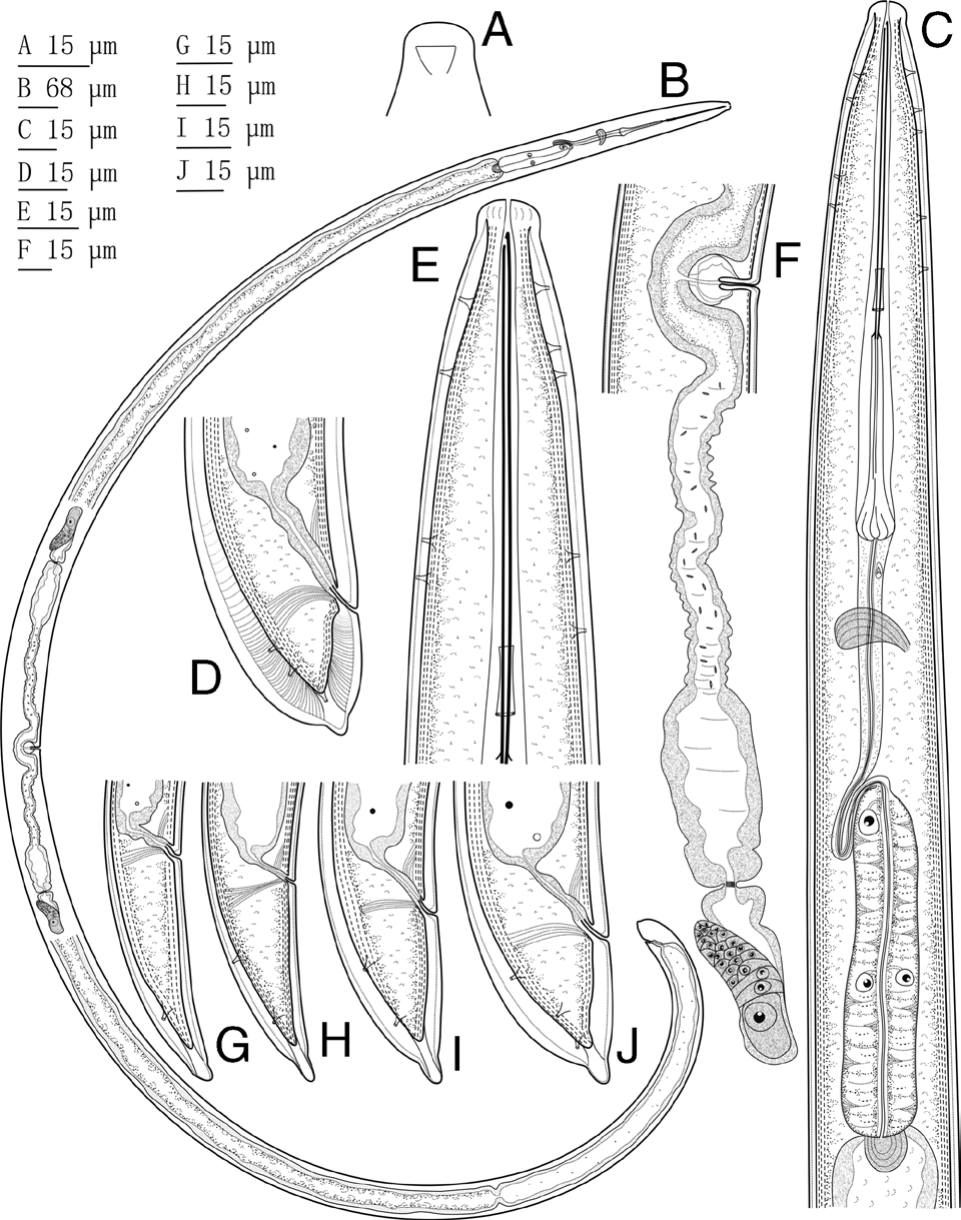
*Xiphinema barooghii* n. sp. (A) Amphidial pouch; (B) Entire body; (C) Neck region; (D) Female tail; (E) Anterior end; (F) Posterior genital branch; (G–J) Tail of juveniles from J1–J4, respectively.

**Figure 2:**
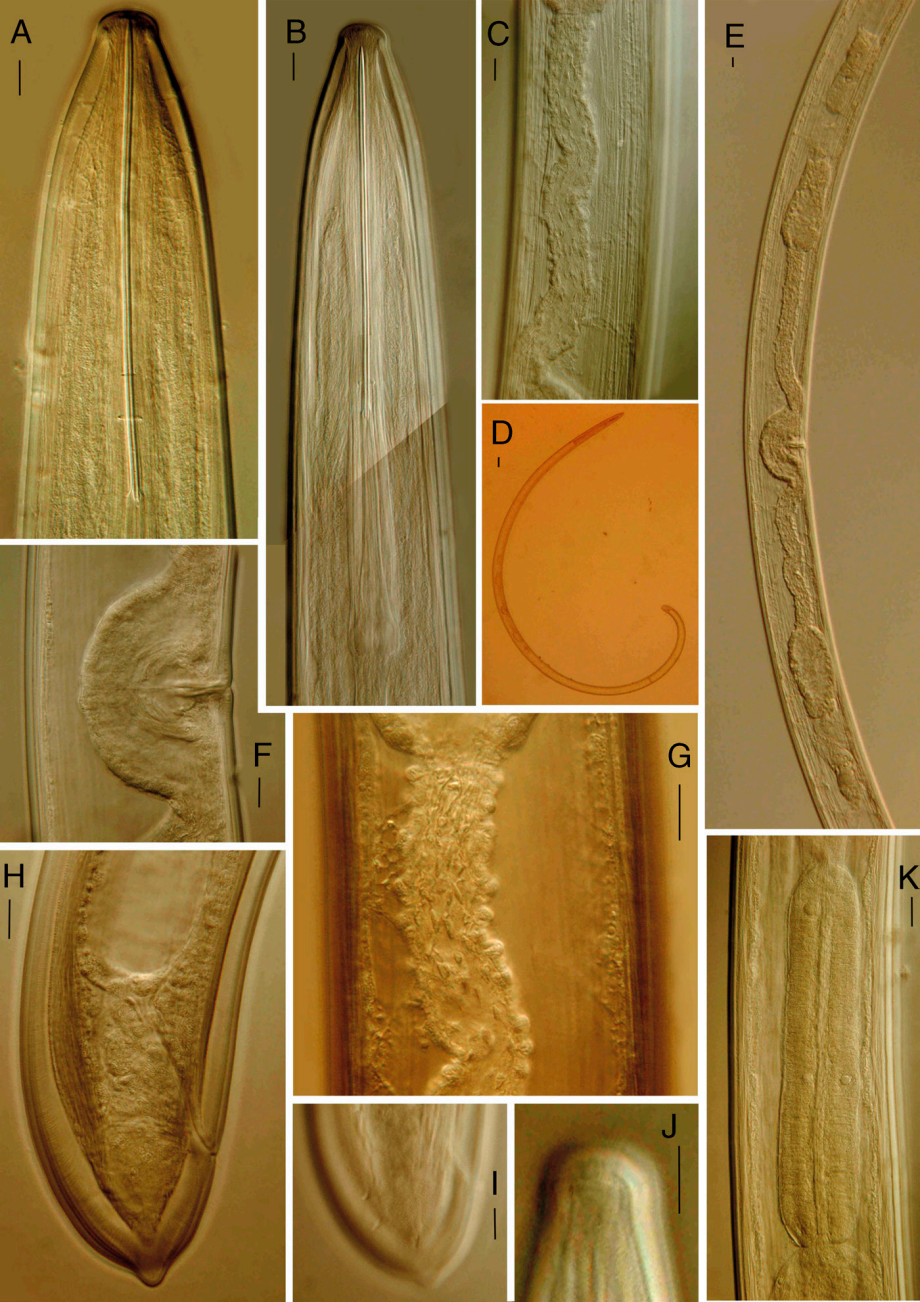
*Xiphinema barooghii* n. sp. Female (A, B) anterior end; (C, G) uterine differentiation spines; (D) entire body; (E) reproductive system; (F) vagina; (H) tail; (I) caudal pores in lateral optical view; (J) amphidial pouch; (K) pharyngeal expansion. (Scale bars: A–K = 10 μm, D = 70 μm).

**Figure 3:**
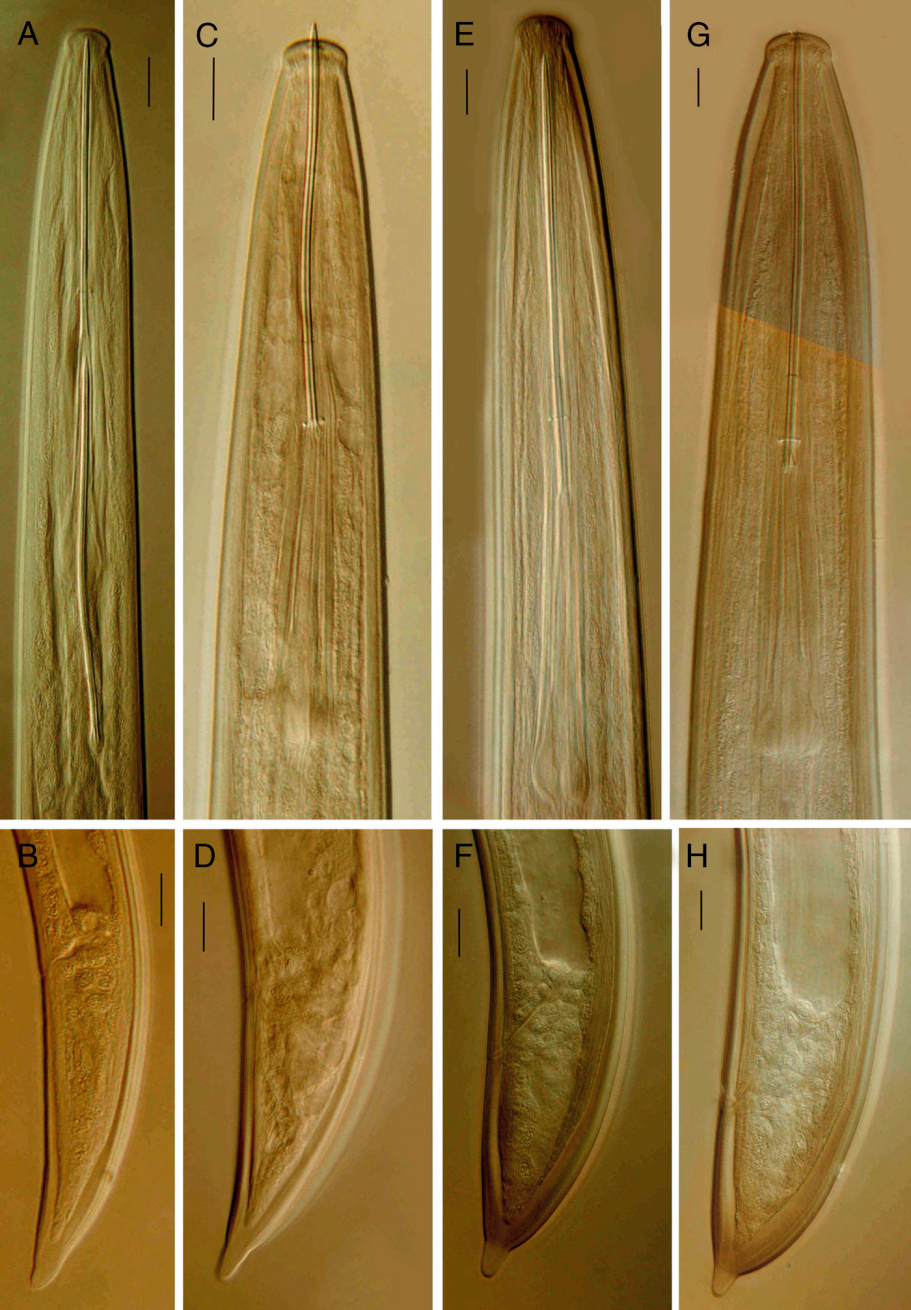
Juvenile stages of *Xiphinema barooghii* n. sp. Anterior region and tail shape of (A, B) first; (C, D) second; (E, F) third and (G, H) fourth juvenile stages, respectively (Scale bars = 10 μm).

*Xiphinema barooghii* n. sp.

(Figs. 1–3; Tables 1–3).

**Table 1:** Morphometrics of *Xiphinema barooghii* n. sp. All measurements are in μm (except L in mm), and in the form: mean ± s.d. (range).

					Female	
Characters	J1	J2	J3	J4	Holotype	Paratypes
n	3	1	5	5	–	8
L	1.15 ± 0.01 (1.14–1.16)	1.73	2.19 ± 0.12 (2.01–2.31)	3.07 ± 0.17 (2.87–3.34)	4.10	4.02 ± 0.21 (3.67–4.25)
a	44.0 ± 2.3 (41.0–47.0)	41	55.0 ± 3.9 (51.0–62.0)	58.0 ± 5.0 (51.0–64.0)	55	59.0 ± 2.4 (53.0–63.0)
b	3.7 ± 0.1 (3.6–3.8)	5.1	5.1 ± 0.3 (4.7–5.6)	6.5 ± 0.6 (5.9–7.5)	8.2	7.7 ± 0.3 (7.0–8.5)
c	18.0 ± 0.7 (17.0–19.0)	29	44.0 ± 2.0 (42.0–47.0)	66.0 ± 6.1 (60.0–76.0)	132	117 ± 10 (103–133)
c′	3.2 ± 0.1 (3.1–3.3)	2.3	1.62 ± 0.04 (1.60–1.70)	1.1 ± 0.1 (1.0–1.3)	0.7	0.80 ± 0.05 (0.70–0.90)
V	–	–	–	–	47	47.0 ± 0.9 (46.0–48.0)
Lip region diam.	10.5 ± 0.3 (10.0–11.0)	10.6	12.2 ± 0.2 (12.0–13.0)	14.0 ± 1.1 (13.0–16.0)	15	14.6 ± 0.3 (14.0–15.0)
Odontostyle length	58.5 ± 0.2 (58.0–59.0)	70	95.0 ± 2.8 (93.0–100.0)	113.0 ± 3.3 (109.0–117.0)	139	136.0 ± 2.7 (132.0–139.0)
Odontophore length	45.0 ± 1.4 (44.0–47.0)	54	63.0 ± 3.0 (60.0–67.0)	76.0 ± 6.3 (70.0–87.0)	82	82.0 ± 3.2 (75.0–85.0)
Spear length	104.0 ± 0.4 (103.0–105.0)	124	158.0 ± 4.8 (150.0–167.0)	189.0 ± 5.7 (181.0–196.0)	221	220.0 ± 3.3 (215.0–225.0)
Replacement odontostyle	72.0 ± 1.6 (71.0–74.0)	93	115.0 ± 2.9 (110.0–119.0)	137.0 ± 1.7 (135.0–139.0)	–	–
Oral aperture to guide ring	51.0 ± 0.5 (50.0–52.0)	65	82.0 ± 3.1 (76.0–86.0)	101.0 ± 2.4 (98.0–104.0)	129	124.0 ± 5.3 (117.0–132.0)
Pharynx length	311.0 ± 3.4 (307.0–315.0)	337	423 ± 27 (396–476)	447 ± 31 (416–484)	500	520 ± 19 (493–553)
Pharyngeal bulb length	88.0 ± 0.7 (87.0–89.0)	88	112.0 ± 8.6 (103.0–125.0)	122.0 ± 2.5 (118.0–125.0)	144	141.0 ± 9.3 (128.0–153.0)
Body diam. at phar. base	24.0 ± 1.6 (23.0–26.0)	37	36.0 ± 3.6 (33.0–41.0)	45.0 ± 4.1 (41.0–51.0)	65	57.0 ± 7.5 (52.0–69.0)
mid-body	24.0 ± 2.1 (24.0–28.0)	42	40.0 ± 3.4 (34.0–44.0)	52.0 ± 5.0 (46.0–59.0)	74	67.0 ± 6.7 (60.0–79.0)
anus	18.0 ± 0.4 (17.0–19.0)	26	30.0 ± 0.8 (29.0–31.0)	40.0 ± 2.7 (36.0–43.0)	41	42.0 ± 1.8 (40.0–44.0)
G1	–	–	–	–	13	12.3 ± 0.4 (12.0–13.0)
G2	–	–	–	–	13	11.0 ± 1.3 (9.0–13.0)
Prerectum length	265 ± 16 (246–284)	305	412 ± 28 (319–444)	572 ± 40 (525–625)	603	595 ± 74 (503–700)
Rectum length	14.0 ± 0.6 (13.0–15.0)	19	26.0 ± 1.4 (22.0–28.0)	33.0 ± 1.5 (31.0–35.0)	49	43.0 ± 5.4 (35.0–51.0)
Tail length	61.0 ± 1.3 (59.0–63.0)	60	49.0 ± 1.1 (48.0–51.0)	46.0 ± 1.5 (44.0–48.0)	30	33.0 ± 2.6 (30.0–38.0)
Hyaline part of tail	8.4 ± 0.3 (8.0–10.0)	13	13.0 ± 1.3 (12.0–15.0)	14.2 ± 0.5 (13.0–15.0)	13	13.0 ± 0.8 (11.0–16.0)

**Table 2:** Specific *α*-numeric code of each *Xiphinema* spp. belonging to X. non-*americanum* morphospecies Group 6 according to **Loof and Luc (1990)**.

*Xiphinema* spp.	A	B	C	D	E	F	G	H	I	J	K	L	References
*spinuterus*	4	3	1	1	4	3	3	2	12	–	–	2	1
*mluci*	4	3	2	123	45	(3)45	23	2	34	2	–	1	1
*xenovariabile*	4	3	23	34	56	23	1	2	3(4)	2	–	2	1
*diannae*	4	3	3	4	45	3	12	2	23	3	–	2	1
*coomansi*	4	3	3	45	456	3	2	2	3	3	–	2	1
*lacrimaspinae*	4	3	4	4	4	3	2	2	3	34	2	1	1
*barbercheckae*	4	3	4	5	56	3	2	2	23	3	–	1	1
*mammatum*	4	3	5a	4	4	3	2	2	3	5	2	2	1
*aequum*	4	3	5a	5	5	45	3	2	3	5	2	2	1
*aceri*	4	3	6a	56	5	4	23	3	3	6	–	1	1
*robbinsi*	4	3	5a	65	56	3(4)	2	2	3(4)	5a	2	2	2
*iranicum*	4	3	5a, b	6	6	4	3(4)	2	3	5	4	1	3
*nuragicum*	4	3	7	6	56	345	3	2	3	7	2	1	4
*pyrenaicum*	4	3	6	6	56	345	3	2	3	6	2	1	4
*adenohystherum*	4	3	7	6	56	45	3	2	3	7	–	1	4
*sphaerocephalum*	4	3	5	6	56	34	3	2	3	5	2	1	4
*mazandaranense*	4	3	7b	6	456	45	2	2	23	7b	2	1	5
*zagrosense*	4	3	6	6	456	45	34	2	3	5	2	1	6
*vuittenezi*	4	3	5(7b)	56	56	34	23	2	3	45	2	1	7
*barooghii* n. sp.	4	3	5a	6	5	4	3	2	3	4	2	1	8

**Notes:** Referenses: 1- Loof and Luc (1990). 2- Pedram et al. (2008). 3- Pedram et al. (2009). 4- Gutiérrez-Gutiérrez et al. (2010). 5- Pedram, Pourjam, Robbins, Ye, Atighi and Decramer (2012). 6- Ghaemi et al. (2012). 7- Barsi et al. (2000). 8- Present paper.

**Table 3:** *Xiphinema* species, locality, associated host and sequences used in this study.

Species	Locality	Host-plant	Accession number
*X. abrantinum*	–	–	AY601625
*X. adenohystherum*	Bollullos par del Condado, Huelva province, Spain	*Vitis vinifera* L.	GU725075
*X. adenohystherum*	Arévalo de la Sierra, Soria province, Spain	Holly tree	KC567164
*X. andalusiense*	Belmez, Cordoba province, Spain	Wild olive	KX244884
*X. baetica*	Manzanilla, Huelva province, Spain	Grapevine	KC567167
*X. bakeri*	–	–	AY601623
*X. barense*	Apulian region, Torre pozzella, Brindisi province, southern Italy	Wild olive (*Olea europaea sylvestris* L.)	KM199690
*X. barense*	Apulian region, Torre Pozzella, Brindisi Province, southern Italy	Wild olive (*Olea europaea sylvestris* L.)	KM199691
*X. barense*	Apulian region, Torre Pozzella, Brindisi Province, southern Italy	Wild olive (*Olea europaea sylvestris* L.)	KM199692
*X. barooghii* n. sp.	Roodghat area, Sufiyan, East-Azarbaijan province, northwest of Iran	Common wheat (*Triticum aestivum* L.)	MH884067
*X. basiri*	–	–	AY601630
*X. belmontense*	Merza, Coruña province, Spain	Pedunculate oak	KC567172
*X. brasiliense*	Shenzhen, China	–	KP793050
*X. brasiliense*	–	–	AY601616
*X. castilloi*	Sefid Rud River near Rasht, Gilan province, Iran	Ash tree	KF446655
*X. cadavalense*	Espiel ,Cordoba province, Spain	Cultivated olive	KX244900
*X. celtiense*	Peñafor, Sevilla province, Spain	Wild olive	KX244889
*X. chambersi*	Florida, USA	–	DQ299512
*X. chambersi*	–	–	AY601617
*X. citricolum*	Florida, USA	–	DQ285668
*X. cohni*	El Puerto de Santa María, Cádiz province, Spain	Stone pine	KC567173
*X. conurum*	Uleila del Campo, Almeria province, Spain	cultivated olive	KX244902
*X. cretense*	Hersonisos, Heraklion province, Crete, Greece	Olive (*Olea europaea* L. subsp. *europaea*)	KJ802878
*X. costaricense*	Pacayitas, La Suiza de Turrialba, Cartago, Costa Rica	Sugarcane	KX931059
*X. coxi*	Hinojos, Huelva province, Spain	Carob tree	KC567175
*X. dentatum*	Czech Republic	Carpinus betulus and Acer platanoides	EU781538
*X. diversicaudatum*	–	–	EF538755
*X. diversicaudatum*	Marchegg, Austria	–	JQ780366
*X. elongatum*	China	–	EF140790
*X. floridae*	Florida, USA	–	DQ299507
*X. gersoni*	Almonte, Huelva province, Spain	Eucalyptus	KC567180
*X. georgianum*	Florida, USA	–	DQ299497
*X. globosum*	Valdeinfierno in the Los Alcornocales Regional Park, Alcalá de los Gazules, Cádiz province, southern Spain	Black alder, *Alnus glutinosa* L. Gaertn., and river bank grapevine, *Vitis riparia*	GU549474
*X. granatum*	Saveh, Markazi province, Iran	Pomegranate trees (*Punica granatum* L.)	JQ240273
*X. hangzhouense*	Hangzhou, Zhejiang Province, China	*Magnolia grandiflora* L.	MF538772
*X. herakliense*	Vathy Rema, Heraklion province, Crete, Greece	Olive tree (*Olea europaea* subsp. *sylvestris*)	KM586345
*X. herakliense*	Vathy Rema, Heraklion province, Crete, Greece	Olive tree (*Olea europaea* subsp. *sylvestris*)	KM586346
*X. herakliense*	Agiofarago, south west Heraklion province, Crete, Greece	Olive	KM586347
*X. herakliense*	Agiofarago, south west Heraklion province, Crete, Greece	Olive	KM586348
*X. herakliense*	Agiofarago, south west Heraklion province, Crete, Greece	Olive	KM586349
*X. herakliense*	Agiofarago, south west Heraklion province, Crete, Greece	Olive	KM586350
*X. herakliense*	Hersonisos, northeast Heraklion province, Crete, Greece	Olive (*Olea europaea* subsp. *europaea* L.)	KM586351
*X. herakliense*	Hersonisos, northeast Heraklion province, Crete, Greece	Olive (*Olea europaea* subsp. *europaea* L.)	KM586352
*X. hispanum*	Andujar, Jaen province, Spain	*Cistus albidus* L.	GU725074
*X. hispidum*	Bollullos par del Condado, Huelva province, Spain	Grapevine (*Vitis vinifera* L.)	HM921346
*X. hunaniense*	Shenzhen, China	–	KP793046
*X. hunaniense*	Shenzhen, China	–	KP793048
*X. index*	Córdoba province, Spain	Grapevine	HM921398
*X. index*	Kentri, Greece	Olive	KJ802882
*X. index*	Córdoba province, Spain	Grapevine	HM921399
*X. index*	Córdoba province, Spain	Grapevine	HM921400
*X. index*	Córdoba province, Spain	Grapevine	HM921401
*X. index*	Cádiz province, Spain	Grapevine	HM921402
*X. index*	Roodghat area, Sufiyan, East-Azarbaijan province, northwest of Iran	Apple (*Malus domestica* L.) variety Red delicious	MH879782
*X. ingens*	Chogha Kaboud village, Harsin, Kermanshah province, Iran	*Astragalus* sp.	KJ956388
*X. insigne*	–	–	AY601619
*X. israeliae*	Roufas, Greece	Olive	KJ802883
*X. israeliae*	Agiofarago, Greece	Wild olive	KJ802884
*X. italiae*	Cabra, Córdoba province, Spain	Grapevine	KC567182
*X. italiae*			AY601613
*X. iznajarense*	Iznaajar, Cordoba province, Spain	Cultivated olive	KX244892
*X. japonicum*	Japan	*Podocarpus macrophyllus* L.	KY131240
*X. laevistriatum*	Florida, USA	–	DQ299505
*X. lambertii*	India	–	HM163211
*X. lupini*	Bollullos par del Condado, Huelva province, Spain	Grapevine (*Vitis vinifera* L.)	HM921352
*X. lupini*	Hinojos, Huelva province, Spain	Grapevine	KC567183
*X. macroacanthum*	Southern Italy	Olive orchards	HF546080
*X. macrodora*	La Granjuela, Córdoba province, Spain	Cultivated olive	KU171040
*X. mengibarense*	Mengibar, Jaen province, Spain	Cultivated olive	KX244894
*X. meridianum*	Sbitla, Kasserine, Tunisia	Cultivated olive	KX062679
*X. naturale*	Florida, USA	–	DQ299515
*X. nuragicum*	Marchena, Seville province, Spain	*Olea europaea* sp. *europaea* L.	GU725071
*X. nuragicum*	Puente Genil, Cordoba province, Spain	*Vitis vinifera* L.	GU725067
*X. oleae*	Tarifa, Cádiz province, Spain	Wild olive	KU171037
*X. poasense*	Toro Amarillo, Valverde Vega, San Carlos Alajuela, Costa Rica	*Eucalyptus*, cypress and fountain grass	MF461347
*X. pseudocoxi*	Alcaracejos, Cordoba province, Spain	Wild olive	KX244915
*X. pyrenaicum*	Cahors, Midi-Pyrenees province, France	*Vitis vinifera* L.	GU725073
*X. rivesi*	Bollullos par del Condado, Huelva province, Spain	Grapevine (*Vitis vinifera* L.)	HM921358
*X. robbinsi*	Sbitla, Kasserine, Tunisia	Cultivated olive	KX062683
*X. robbinsi*	Abida, Kairouan, Tunisia	Cultivated olive	KX062685
*X. santos*	–	–	AY601587
*X. savanicola*	–	–	AY601620
*X. setariae*	–	–	AY601621
*X. sphaerocephalum*	Coto Rios, Jaen province, Spain	*Quercus faginea* L.	GU725076
*X. tarjanense*			DQ299511
*X. tica*	Chirraca, San Ignacio de Acosta, San José, Costa Rica	Grapevine	KY623485
*X. turcicum*	Sanlúcar de Barrameda, Cádiz province, Spain	Grapevine	KC567185
*X. turdetanensis*	Sanlúcar de Barrameda, Cádiz province, Spain	Stone pine	KC567186
*X. vuittenezi*	Czech Republic	–	EF614266
*X. vuittenezi*	–	–	AY601614
*X. vulgare*	Florida, USA	–	DQ299514
*X. zagrosense*	Madavan village, Kohgiluyeh and Boyer-Ahmad province, Iran	Grasses	JN153101

### Description

#### Female

It is characterized by having a cylindrical body, gradually tapered towards both ends, ventrally curved, open C to G-shape upon fixation. It has a two-layered cuticle and very fine transverse striations are visible more in tail region, 3.0‒4.0 μm wide at mid-body and 11‒16 μm at the tail tip. Lateral pores are present along the body, with four dorsal and five ventral located between anterior end and guiding ring. Lateral chords of 14‒17 μm or those occupying one-fifth of the mid-body diameter are present. The lip region is flat and the cephalic region is rounded, separated from body contour by a shallow depression, 1.7‒2.5 times as broad as high and one-fourth to one-fifth (21–28%) of body diameter at neck base. Amphidial fovea is cup shaped, with aperture occupying 52‒59% of the corresponding lip region diameter, located slightly anterior to depression of head, remainder of body and pouches typical of the genus. Odontostyle is long and slender, furcates at junction with odontophore, 8.8‒9.4 times lip region diameter or 1.5‒1.8 times odontophore length. Odontophore with well-developed basal flanges, 14‒18 μm wide, exists. A double guiding ring and a guiding sheath of 3‒30 μm length, depending on the degree of protraction/retraction of stylet, is present. Esophagus is slender with a weak muscular narrow part extending to a cylindrical terminal esophageal bulb with three nuclei. The esophageal basal bulb is 128‒153 μm long, occupying about 24‒29% of total esophagus length and 24‒31 μm width. The nucleus of dorsal esophageal gland (DN) is located at the beginning of basal bulb (8‒11%), 3.7‒5.1 μm in diameter, dorsal gland esophageal orifice (DGEO), 4.7‒6.5 μm from anterior end, and two smaller ventrosublateral nuclei (SVN) located at 52‒57% of the terminal bulb length. The esophageal intestinal valve is rounded conoid with 12‒14 μm length. The tip of reserve odontostyle (vestigium) is observed in isthmus in some specimens. The nerve ring is positioned at 55 68% length from anterior end and intestine is simple. The female reproductive system is didelphic‒amphidelphic with equally developed genital branches (428–575) μm and (387–512) μm long, respectively. Each branch is composed of a 64–117 μm long reflexed ovary, not reaching the oviduct‒uterus junction; oocytes are arranged first in several rows and then in a single row; oviduct is 70–160 μm long with developed *pars dilatata oviductus* near the sphincter, joining the ovary terminally; oviduct-uterus junction is marked by a poorly developed sphincter and a 256–297 μm long bipartite uterus composed of *pars dilatata uteri* close to sphincter and a tubular part containing spines 2–5 μm long, spindle-shaped and scattered between the enlarged distal portion and the ovejector, with a lack of sperm in the genital tract. Ovejector is well developed (71–84 × 15–28 μm); vagina is perpendicular to body axis, 31–37 μm long or 42–52% of corresponding body diameter in lateral view and surrounded by robust muscles. Vulva, a transverse slit, pre-equatorial in position, is present. Pre-rectum variable is 503‒700 μm in length and the rectum length is 0.7‒1.1 times anal body diameter. A short tail, conoid and dorsally convex, ventrally directed with a small terminal peg, 4.5‒7.5 μm long, and a distinct terminal blind canal, exists. Three to four caudal pores are present on each side.

#### Male

Not found.

#### Juveniles

**Figure 4:**
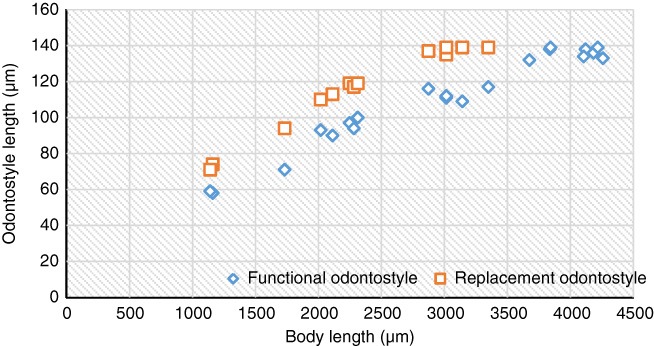
*Xiphinema barooghii* n. sp. Graph of correlation of functional and replacement odontostyle to body length in all developmental stages from Jl to mature females.

All four juvenile stages were identified using morphological characters such as body length, length of replacement and functional odontostyle (Robbins et al., 1996). The scatter diagram representing the relationships between body length, functional and replacement odontostyle of females and juveniles is given in Figure 4. Juveniles are similar to adults in gross morphology, except for their smaller size, longer tail, and undeveloped reproductive organs. Jl is characterized by the lip region being separated from body contour by a deep depression, replacement odontostyle tip being close to base of functional odontostyle and located at the level of odontophore, and tail conoid and dorsally convex, directed ventrally, has a depression on dorsal and ventral sides at hyaline level, with a curved finger like cuticular extension and blind canal at the end. The lip region in J2 is separated from body contour by a depression but in J3 and J4, it is similar to that of female, *i.e.,* flat with the cephalic region rounded and separated from body contour by a shallow depression. In J2‒J4, replacement odontostyle is located at some distance from odontophore. In J2, the tail is conoid and dorsally convex, slightly bent ventrally and with a dorsal depression at hyaline region level; in J3, tail is conoid, dorsally convex, ventrally more or less flat with a slightly developed mucro and tail of J4 is similar to that of female.

#### Diagnosis and relationships

*Xiphinema barooghii* n. sp. belongs to morphospecies group 6 *sensu* Loof and Luc (1990). It is an apparently parthenogenetic species characterised by a medium-to-moderate long body of 3.67–4.25 mm; a C to G-shape upon fixation; a flat lip region, a rounded cephalic region, separated from body contour by a shallow depression of 14–15 μm width; along and slender odontostyle having 132–139 μm length; a guide ring being located at 117–132 μm from anterior end; a female reproductive system being didelphic with two opposite almost equally developed reproductive branches with spines in the tubular part of the uterus; a short tail, conoid and dorsally convex, ventrally directed with a small terminal peg, 4.5–7.5 μm long, and a distinct terminal blind canal; lack of males in population, having four juvenile stages and J1 tail having a depression on dorsal and ventral sides at hyaline level, with a curved finger like cuticular extension at the end.

The identification codes for the new species, according to the polytomous key of Loof and Luc (1990), are: A4, B3, C5a, D6, E5, F4, G3, H2, I3, J4, K2, L1.

Based on the molecular and morphological similarities, the new species is closely related to *X. aceri* (Chizhov et al., 1986); *X*. *granatum* (Pedram, Pourjam, Palomares-Rius, Ghaemi, Cantalapiedra-Navarrete and Castillo, 2012); *X. herakliense* (Tzortzakakis et al., 2015); *X. zagrosense* (Ghaemi et al., 2012), and *X. vuittenezi* (Luc et al., 1964), but it can be separated using the morphometric data, characters of the juveniles, especially the shape of their tail in the first stage and partial sequences of 28S rDNA (except *X. aceri* as it currently lacks molecular data of D2–D3 expansion part of 28S rDNA).

Compared to *X. aceri,* the new species has a shorter body (3.67–4.25 vs 4.90–5.50 mm), smaller a (53–63 vs 75–83) and V values (46–48 vs 48–51), shorter odontophore (75–85 vs 83–101 μm) and juvenile characters as well, e.g., shorter tail length (59–63 vs 67.2–68.8 μm) and tail characters in J1. *Xiphinema barooghii* n. sp. differs mainly from *X*. *granatum* by having lower a (53–63 vs 74–99), b (7.0–8.5 vs 8.5–11.0) and c′ (0.7–0.9 vs 1.2–1.5) values, longer odontostyle (132–139 vs 118–132 μm), odontophore (75–85 vs 65–74 μm) and spear (215–225 vs 189–204 μm), esophagus (493–553 vs 360–460 μm) and esophageal basal bulb length (128–153 vs 77–104 μm), posteriorly located guiding ring (117–132 vs 100–116 μm), absence of males, uterus with spines vs devoid of any *Z*-differentiation or spines and juvenile characters. In addition, *X. barooghii* n. sp. is similar to *X. herakliense* but differs by a longer esophageal basal bulb (128–153 vs 94–121 μm), absence vs presence of a pseudo-Z-organ and crystalloid bodies in the uterus. Furthermore, *X. herakliense* is an amphimictic species and has functional males and sperm in the female reproductive system compared to the absence of males in the new species. The new species differs from *X. zagrosense* by a shorter odontostyle (132–139 vs 151–169 μm), odontophore (75–85 vs 94–105 μm) and spear length (215–225 vs 246–274 μm), slightly smaller lip region width (14–15 vs 15–18 μm), the shape of tail (conoid, dorsally convex, ventrally directed with a small terminal peg and distinct terminal blind canal vs conoid and dorsally convex, with rounded end lacking a mucro or cuticular projection) and differences in juvenile characters. Finally, *X. barooghii* n. sp. can be differentiated from *X. vuittenezi* (according to original description) by a slightly longer body in the females (3.67–4.25 vs 2.63–3.83 mm), longer spear (215–225 vs 183–212 μm), wider body diameter at neck base (52–69 vs 44 μm) and mid-body or vulva level (60–79 vs 39–58 μm), cuticle two vs three layered, cephalic region separated from body contour by a shallow depression and 21–28% of body diameter at neck base vs clearly depression and 31% of body diameter at neck base, greater length and width of esophageal basal bulb, 128–153 × 24–31 vs 109–126 × 18–23 μm, cardia 12–14 μm long and rounded conoid vs 6.5 μm (calculated from the image) conoid. In addition, there are a number of differences in juvenile characters, in J1: greater body (1.14–1.16 vs 0.78–1.03 mm), odontostyle (58–59 vs 47–53 μm), odontophore (44–47 vs 34–40 μm), spear (103–105 vs 81–93 μm), replacement odontostyle (71–74 vs 62–71 μm) length, oral aperture to guide ring (50–52 vs 43–50 μm), tail length (59–63 vs 40–52 μm) and shape (presence a depression on dorsal and ventral sides at hyaline level, with a curved finger like cuticular extension at the end vs absence); in J2: longer body (1.73 vs 1.07–1.49 mm), odontophore (54 vs 42–50 μm), spear (124 vs 107–121 μm), replacement odontostyle (93 vs 80–87 μm), oral aperture to guide ring (65 vs 56–59 μm) and tail length (60 vs 43–50 μm), larger body diam. at mid-body (42 vs 22–34 μm) at anus level (26 vs 15–23 μm); in J3: higher body length (2.01–2.31 vs 1.47–1.95 mm), odontostyle (93–100 vs 79–89 μm) and spear (150–167 vs 130–146 μm) length and a value (51–62 vs 37–48); in J4: longer body (2.87–3.34 vs 2.01–2.74 mm), replacement odontostyle (135–139 vs 121–135 μm) and a value (51–64 vs 44–50).

#### Type habitat and locality

Soil samples were collected from the rhizosphere of common wheat (*Triticum aestivum* L.) in Roodghat area, Sufiyan, East-Azarbaijan province, northwest of Iran, during 2016 and 2017 (GPS coordinates: N 38°22′ 10″, E 46° 07′ 26″, altitude 1808 m a.s.l.).

#### Type material

Holotype and paratype females and juveniles were deposited at Nematology Collection of the Department of Plant Protection, Faculty of Agriculture, University of Tabriz, Tabriz, Iran. Two paratype females were also deposited at Nematode Collection of the University of Jaen, Spain.

#### Etymology

The new species is named in honor of Dr. Hassan Barooghi, the late Entomologist and Associate Professor in Department of Plant Protection, University of Tabriz, Tabriz, Iran.

#### Molecular characterization and phylogeny

**Figure 5:**
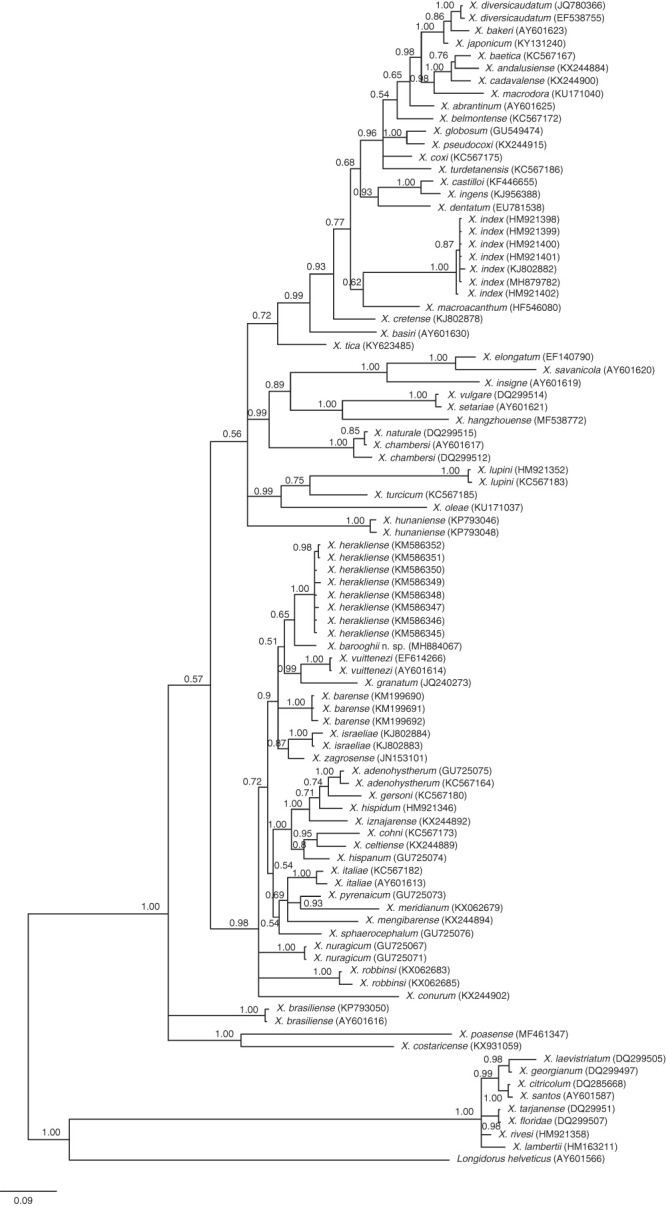
Bayesian tree inferred under the GTR + I + G model from 28S rDNA D2–D3 expansion domains of *X. barooghii* n. sp. and *X. index* (−lnL = 9,085.8555; AIC = 18,191.7109; freqA = 0.2402; freqC = 0.2341; freqG = 0.2885; freqT = 0.2373; *R*(a) = 1.0503; *R*(b) = 2.8325; *R*(c) = 2.6784; *R*(d) = 0.5047; *R*(e) = 4.0878; *R*(f) = 1.0000). Posterior probabilities are given for appropriate clades. Newly obtained sequences are indicated by bold letters.

For molecular analysis, one D2–D3 28S rDNA sequence, 800 bp long, was obtained (GenBank accession no. MH884067). The evolutionary relationships of the new species, *Xiphinema barooghii* n. sp., are shown in Figure 5. The tree is reconstructed from 93 sequences, out of which 84 sequences belong to species of *Xiphinema* non-*americanum* group, 8 from *X. americanum* group and *Longidorus helveticus* sequence as out group taxon. The *X.* non-*americanum* species included in the analysis had representatives from all morphogroups as defined by Loof and Luc (1990). The species from GenBank with the highest match in Nblast search with *X. barooghii* n. sp. were selected for phylogenetic analysis and the new species showed 98, 97, 97, 96, 95, 94 and 91 percent of similarity to *X. herakliense* (KM586348), *X. zagrosense* (JN153101), *X. israeliae* (KJ802883), *X. barense* (KM199691), *X. vuittenezi* (EF614266), *X*. *granatum* (JQ240273) and *X. robbinsi* (KX062685), respectively, and 15, 20, 22, 29, 30, 41 and 53 nucleotides differences, respectively, as compared to the new species. The average nucleotide composition is as follows: 24.02% A, 23.41% C, 28.85% G and 23.73% T.

*Xiphinema barooghii* n. sp. is phylogenetically related to *X. herakliense, X*. *granatum* and *X. vuittenezi* from morphospecies Groups 5, 6 and 8, well positioned within, but clearly separated from them. In this subclade, all four species share a conoid and dorsally convex female tail with a central subdigitate peg. In this regard, our data did not demonstrate a correlation between morphospecies and their grouping in phylogenetic analysis using molecular markers, confirming the findings by Gutiérrez-Gutiérrez et al. (2013), Roshan-Bakhsh et al. (2014), De Luca et al. (2014), and Tzortzakakis et al. (2015).

In summary, molecular characterisation and phylogenetic analysis of D2–D3 region sequence and morphological and morphometric analyses clearly supported the status of *Xiphinema barooghii* n. sp. as a new taxon within the *X.* non-*americanum* group.

### *Xiphinema index* Thorne and Allen (1950)

**Figure 6:**
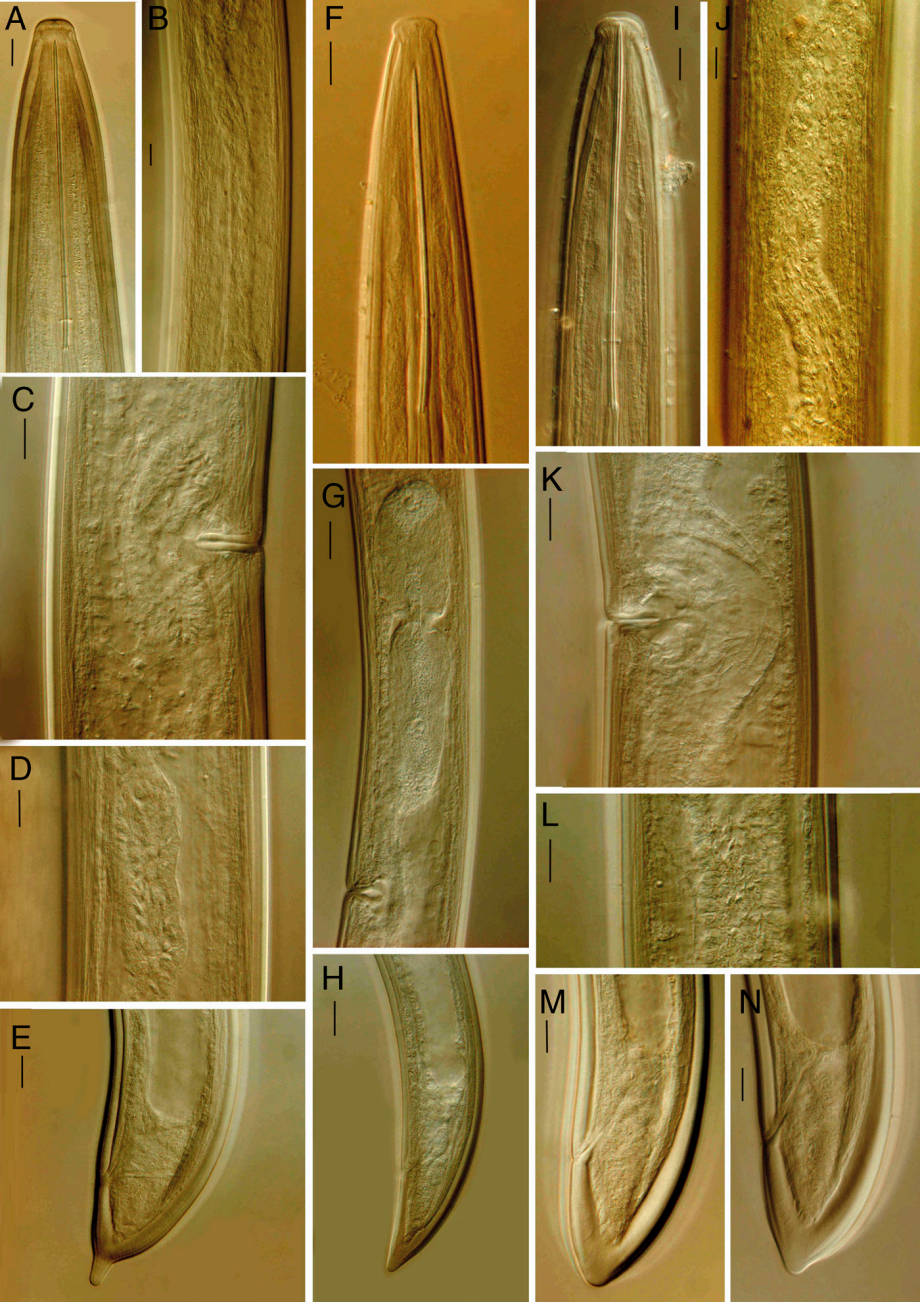
(A–E) *Xiphinema index* Thorne and Allen, 1950, (A) Anterior end; (B) Tubular part of uterus; (C) Vagina; (D) Pars dilatata oviductus; (E) Tail, (F–H) *X. pachtaicum* (Thlaganov, 1938) Kirjanova, 1951, (F) Anterior end; (G) Anterior genital branch; (H) Tail, (I–N) *X. vuittenezi* Luc, Lima, Weischer and Flegg, 1964, (I) Anterior end; (J, L) Uterine differentiation spines; (K) Vagina; (M, N) Tail. (Scale bars = 10 μm).

(Fig. 6 A–E; Tables 3, 4)

**Table 4:** Morphometrics of the Iranian populations of *Xiphinema* species. All measurements are in μm (except L in mm), and in the form: mean ± s.d. (range).

	***X. index***	***X. pachtaicum***	***X. vuittenezi***
Characters	Female	Female	Female
n	5	5	6
L	2.98 ± 0.18 (2.68–3.13)	2.04 ± 0.08 (1.95–2.17)	3.51 ± 0.16 (3.25–3.76)
a	55.0 ± 4.2 (50.0–60.0)	61.0 ± 8.7 (52.0–74.0)	69.0 ± 5.0 (63.0–78.0)
b	6.7 ± 0.3 (6.3–7.3)	6.7 ± 0.6 (6.3–7.8)	7.6 ± 0.1 (7.3–7.9)
c	76.0 ± 2.4 (74.0–80.0)	69.0 ± 3.6 (64.0–73.0)	89.0 ± 3.6 (77.0–95.0)
c′	1.04 ± 0.05 (1.00–1.10)	1.50 ± 0.10 (1.40–1.70)	1.10 ± 0.08 (1.00–1.20)
V	37.0 ± 0.7 (36.0–38.0)	54.0 ± 1.2 (53.0–56.0)	49.0 ± 0.4 (47.0–50.0)
Lip region diam.	13.0 ± 0.8 (12.0–14.0)	9.4 ± 0.5 (9.0–10.0)	13.0 ± 0.7 (12.0–14.0)
Odontostyle length	121.0 ± 2.7 (118.0–125.0)	83.0 ± 2.6 (81.0–87.0)	122.0 ± 3.7 (118.0–128.0)
Odontophore length	70.0 ± 1.7 (69.0–73.0)	50.0 ± 3.3 (44.0–54.0)	68.0 ± 4.1 (51.0–75.0)
Spear length	192.0 ± 2.7 (189.0–195.0)	133.0 ± 2.7 (130.0–137.0)	191.0 ± 6.8 (175.0–200.0)
Oral aperture to guide ring	114.0 ± 4.3 (106.0–119.0)	70.0 ± 3.6 (68.0–77.0)	108.0 ± 4.3 (100.0–113.0)
Pharynx length	437 ± 11 (421–450)	303 ± 20 (250–328)	457 ± 11 (443–475)
Pharyngeal bulb length	99.0 ± 6.4 (87.0–110.0)	77.0 ± 4.6 (72.0–84.0)	114 ± 10 (100–131)
Body diam. at phar. base	47.0 ± 3.4 (43.0–52.0)	29.0 ± 2.2 (26.0–32.0)	41.0 ± 2.7 (37.0–45.0)
mid-body	54.0 ± 3.8 (45.0–59.0)	33.0 ± 2.4 (28.0–37.0)	49.0 ± 5.3 (41.0–56.0)
anus	35.0 ± 3.5 (31.0–40.0)	19.0 ± 1.4 (17.0–21.0)	35.0 ± 2.1 (32.0–38.0)
G1	13.2 ± 0.4 (13.0–14.0)	12.0 ± 1.2 (11.0–14.0)	12.6 ± 0.3 (12–13)
G2	12.0 ± 1.3 (11.0–14.0)	12.0 ± 1.9 (10.0–14.0)	12.0 ± 0.8 (11.0–13.0)
Prerectum length	320 ± 46 (250–381)	168 ± 17 (134–187)	496 ± 58 (431–575)
Rectum length	30.0 ± 1.9 (27.0–32.0)	24.0 ± 3.5 (23.0–28.0)	31.0 ± 1.2 (29.0–33.0)
Tail length	39.0 ± 0.9 (36.0–41.0)	29.0 ± 1.1 (28.0–31.0)	39.0 ± 1.4 (38.0–41.0)
Hyaline part of tail	17.0 ± 1.4 (15.0–19.0)	10.0 ± 0.8 (9.0–11.0)	14.0 ± 1.8 (12.0–16.0)

#### Distribution

Iran, East-Azarbaijan province, Sufiyan, Roodghat area (GPS coordinates: N 38° 19′ 59″ E 46° 07′ 00″, altitude 1582 m a.s.l.), in the rhizosphere of apple (*Malus domestica* L.) variety Red delicious.

#### Remarks

*Xiphinema index* is a soil dweller, its major economic host is grapevine and acts as vector of *Grapevine Fanleaf Virus* (GFLV), very well known, worldwide spread species, recorded from many countries of Africa, America, Australia, Asia and Europe. In Iran, this species was first observed by Mojtahedi et al. (1980) in cultivated soils and in natural woodland as well and then reported from different parts of Iran. Comparison of our population sequence with the GenBank database showed that Iranian *X. index* (MH879782) has 99% identity with other populations of the species. Phylogenetic analysis placed our population with other populations of *X. index* in a clade with 0.87 PP values. Males were not found but a few females contain sperm cells in their ovejector and *pars dilatata oviductus*. This Iranian population of females of *X. index* is within the morphometrical ranges recorded for the species (for comparative purposes, see Thorne and Allen, 1950; Mojtahedi et al., 1980; Lamberti et al., 1987; Barsi and Lamberti, 2000a; Jawhar et al., 2006; Gutiérrez-Gutiérrez et al., 2011; Meza et al., 2012).

### *Xiphinema pachtaicum* (Tulaganov, 1938; Kirjanova, 1951)

(Fig. 6 F–H; Table 4)

#### Distribution

It was collected in 2016 and 2017 from Iran, East-Azarbaijan province, Sufiyan, Roodghat area (GPS coordinates: N 38°22′ 10″, E 46° 07′ 26″, altitude 1808 m a.s.l.), from the rhizosphere of common wheat (*Triticum aestivum* L.).

#### Remarks

This species is widely distributed in the rhizosphere of different plants in agricultural lands and reported from several localities in the world including Africa, America, Australia, Asia and Europe. Little is known about its role as a plant pathogen and it has not been recorded as a vector of plant viruses. Mojtahedi et al. (1980) reported *X. pachtaicum* for the first time from vineyards in Iran; later on, it was obtained from different localities in the country from the rhizosphere of different plants. The Iranian specimens fit, morphologically and morphometrically, well to earlier descriptions of the species (Fadaei et al., 2003; Getaneh et al., 2015; Lazarova et al., 2016).

### *Xiphinema vuittenezi* Luc et al. (1964)

(Fig. 6 I–N; Table 4).

#### Distribution

It was collected from Iran, East-Azarbaijan province, Sufiyan, Roodghat area (GPS coordinates: N 38° 22′ 10″ E 46° 07′ 26″, altitude 1481 m a.s.l.), from the rhizosphere of common wheat (*Triticum aestivum* L.).

#### Remarks

This species is widely spread in Europe. Besides, it has been recorded from Asia, North America, South America and Australia. It inhabits vineyards and various orchards (Andrássy, 2009). The original description of *X. vuittenezi* did not consider the presence of spines in the uteri and such structures were not mentioned in the revised polytomous key to the species of *Xiphinema* by Loof and Luc (1990), although distinct spindle-shaped structures of variable size and number were observed in the tubular portion of the uterus of an Iranian population of *X. vuittenezi* by Mojtahedi et al. (1980). For the first time in Iran, this species has been reported by Mojtahedi et al. (1980) from different regions of the country. The present Iranian specimens correspond well with the earlier descriptions in their general morphology and morphometrics as the relevant measurements and values are totally coincident or widely overlapping (Luc et al., 1964; Barsi and Lamberti, 2000b; Kumari and Decraemer, 2006).
